# Cortical Hub for Flavor Sensation in Rodents

**DOI:** 10.3389/fnsys.2021.772286

**Published:** 2021-11-15

**Authors:** Chad L. Samuelsen, Roberto Vincis

**Affiliations:** ^1^Department of Anatomical Sciences and Neurobiology, University of Louisville, Louisville, KY, United States; ^2^Department of Biological Science and Program in Neuroscience, Florida State University, Tallahassee, FL, United States

**Keywords:** flavor, chemosensory, multimodal, cortex, olfaction, gustation, somatosensation

## Abstract

The experience of eating is inherently multimodal, combining intraoral gustatory, olfactory, and somatosensory signals into a single percept called flavor. As foods and beverages enter the mouth, movements associated with chewing and swallowing activate somatosensory receptors in the oral cavity, dissolve tastants in the saliva to activate taste receptors, and release volatile odorant molecules to retronasally activate olfactory receptors in the nasal epithelium. Human studies indicate that sensory cortical areas are important for intraoral multimodal processing, yet their circuit-level mechanisms remain unclear. Animal models allow for detailed analyses of neural circuits due to the large number of molecular tools available for tracing and neuronal manipulations. In this review, we concentrate on the anatomical and neurophysiological evidence from rodent models toward a better understanding of the circuit-level mechanisms underlying the cortical processing of flavor. While more work is needed, the emerging view pertaining to the multimodal processing of food and beverages is that the piriform, gustatory, and somatosensory cortical regions do not function solely as independent areas. Rather they act as an intraoral cortical hub, simultaneously receiving and processing multimodal sensory information from the mouth to produce the rich and complex flavor experience that guides consummatory behavior.

## 1. Introduction

Eating is a multisensory experience (Small et al., 2004; Small, 2012; Prescott, 2015; Spence, 2015). While extraoral sensory cues (e.g., orthonasal smell, sight, and food-related sounds) can influence food intake, the perception of flavor originates from core sensations inside the mouth. When a food or beverage enters the oral cavity, multiple sensory modalities are engaged simultaneously. The chewing, movement, and swallowing of food activates somatosensory receptors (e.g., tactile, thermal, proprioceptive, and nociceptive) located throughout the oral cavity and contributes to the release of volatile molecules (i.e., odorants) that travel retronasally via the oropharynx to activate olfactory receptors in the nasal epithelium. Meanwhile, non-volatile chemicals (i.e., tastants) dissolve in the saliva to activate taste receptors primarily located in the tongue. Largely, this sensory information is transmitted centrally along separate pathways, but the integration of these three intraoral senses into a unitary object generates the perception of flavor (Small, 2012). As a consequence, the intraoral sensations associated with eating are inherently related (Schul et al., 1996; Sakai and Yamamoto, 2001; Sakai and Imada, 2003; Torregrossa et al., 2012; Blankenship et al., 2019; Fredericksen et al., 2019; Elliott and Maier, 2020; Maier and Elliott, 2020; McQueen et al., 2020). Our current understanding of the behavioral and neural relationships between the intraoral senses is due in large part to many notable human studies (Arabie and Moskowitz, 1971; Moskowitz, 1973; Bartoshuk et al., 1982; Frank et al., 1993; Stevenson et al., 1995; Dalton et al., 2000; Hollowood et al., 2002; De Araujo et al., 2003; de Araujo et al., 2013; Prescott et al., 2004; Small et al., 2004, 2008; Veldhuizen et al., 2010; Lim and Johnson, 2011; Veldhuizen and Small, 2011; Green et al., 2012). Yet, the circuit mechanisms remain unclear. Recent findings from animal model studies are beginning to elucidate the neural substrates underlying the multimodal processing of flavor.

One key question is at which level of the bottom-up sensory pathways do all three intraoral components of flavor converge? For a subset of somatic stimuli (i.e., temperature), there is evidence for taste-somatosensory interactions as early as the peripheral taste-receptor cell (Talavera et al., 2005). Additionally, gustatory and somatosensory signals appear to interact at different subcortical areas along the taste pathway (Beidler, 1954; Nagaki et al., 1964; Sato, 1967; Ogawa et al., 1988; Travers and Norgren, 1995; Verhagen et al., 2003; Breza et al., 2006; Wilson and Lemon, 2013; Li and Lemon, 2019). However, there is little evidence of direct subcortical interactions with the olfactory system. Although some neurons in the rat's nucleus of the solitary tract (NST) (Van Buskirk and Erickson, 1977; Escanilla et al., 2015) and parabrachial nucleus (PBN) (Di Lorenzo and Garcia, 1985) are modulated by odors, the source of these olfactory signals is likely due to cortico-fugal projections rather than direct input from the olfactory bulb (Escanilla et al., 2015). Given the findings of a number of recent anatomical and physiological studies, the more commonly held view is that convergence and integration of all three intraoral modalities likely occurs at the level of cortex (Small, 2012).

Therefore, we focus this review on findings gleaned from studies in rodents, concentrating on the cortical areas known to process sensory information arising from the mouth. First, we describe the anatomical features of the three primary sensory cortical regions subtending flavor sensation (i.e., piriform cortex, gustatory cortex, and somatosensory cortex), paying particular attention to studies examining the direct corticocortical connectivity between them. Second, we review neurophysiological findings detailing how the three cortical regions represent and process their unimodal component of flavor. Lastly, we discuss the evidence from recent studies highlighting the capacity of the three sensory cortical regions to process multimodal information related to flavor. Throughout this review, we will emphasize the critical gaps in knowledge that require further investigation to better understand the neural substrates underlying the multimodal processing of flavor.

## 2. Anatomy and Connectivity of the Three Primary Sensory Cortical Regions

**Figure 1:**
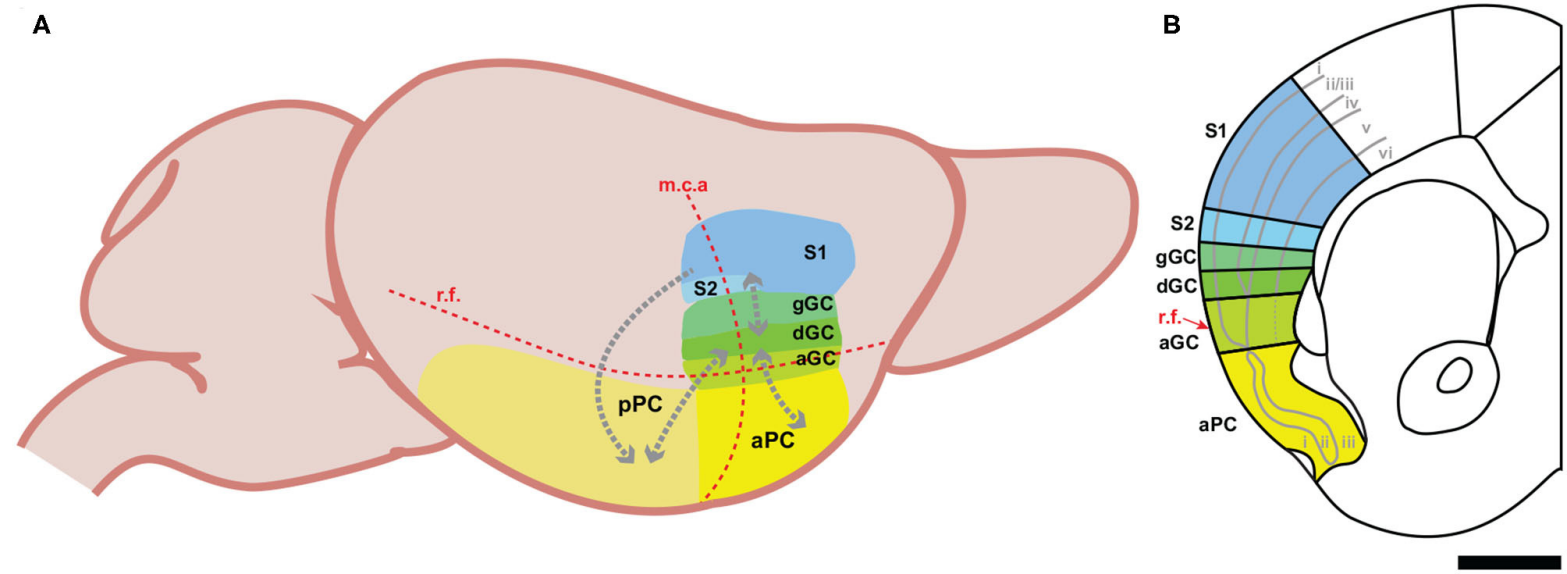
Schematic representations of the three intraoral cortical regions in the mouse. **(A)** A lateral view displaying the relationship between the oral-somatosensory cortex, gustatory cortex, and olfactory cortex. The red dotted lines indicate the position of two key landmarks: the rhinal fissure (r.f.) and the middle cerebral artery (m.c.a.). The gray dotted arrows represent the corticocortical connections between regions. aGC, agranular gustatory cortex; dGC, dysgranular gustatory cortex; gGC, granular gustatory cortex; mca, middle cerebral artery; aPC, anterior piriform cortex; pPC, posterior piriform cortex; rf, rhinal fissure; S1, somatosensory cortex, area 1; S2, somatosensory cortex; area 2. **(B)** A coronal section of the mouse brain containing the oral-somatosensory cortex, gustatory cortex, and piriform cortex. The solid gray lines indicate the divisions between the cortical layers. Note the loss of layer 4 in the dysgranular gustatory cortex. The dotted gray line in the agranular gustatory cortex represents the fading boundary between layer 5 and layer 6, while the red arrow highlights the anatomical position of the rhinal fissure (r.f.). Black bar is 1 mm.

Volatile chemicals, *odorants or odors*, reach olfactory sensory neurons localized in the main olfactory epithelium via two routes. Orthonasal olfaction occurs when odors are inhaled through the nares directly into the nasal cavity (e.g., when smelling a flower). Retronasal olfaction occurs when odors travel from the mouth, passing through the orophyranyx, activate olfactory receptors in the nasal epithelium (Rozin, 1982; Masaoka et al., 2010; Gautam and Verhagen, 2012). Although olfactory signals generated by either route are transmitted to the main olfactory bulb by cranial nerve I, retronasal olfaction is a key component for the perception of flavor (Small, 2012; Small and Green, 2012; Bartoshuk et al., 2019). The olfactory system is unique among the senses because sensory signals reach the cortex prior to being processed by the thalamus (Shepherd, 2005). In rodents, output neurons from the main olfactory bulb project to a number of cortical areas important for olfactory-dependent behaviors, including the anterior olfactory nucleus (Brunjes et al., 2005), olfactory tubercle (Wesson and Wilson, 2011), entorhinal cortex (Witter et al., 2017), and piriform cortex (Haberly and Price, 1977; Igarashi et al., 2012). Often called the primary olfactory cortex, the piriform cortex is located on the ventrolateral surface of the brain (Figure 1), immediately ventral to the insular cortex, and receives the majority of projections from the main olfactory bulb (Ghosh et al., 2011). Phylogenetically one of the oldest cortical structures, the piriform cortex is a three-layered paleocortex (Rowe and Shepherd, 2016). Layer I contains the apical dendrites of the pyramidal neurons in piriform cortex, the axons of projection neurons from the olfactory bulb, and corticocortical association fibers, layer II consists primarily of pyramidal cell bodies, and layer III is composed of deep pyramidal cells, pyramidal cell basal dendrites, a variety of interneurons, and is densely innervated by corticocortical association fibers (Haberly, 2001; Neville and Haberly, 2004). In rodents, the piriform cortex is traditionally divided into functionally distinct anterior (aPC) and posterior (pPC) subregions due to differences in their cytoarchitecture, connectivity, and representation of olfactory signals (Wilson and Sullivan, 2011). The anterior piriform cortex is densely innervated by projections from the main olfactory bulb, while projections from the bulb are reduced and supplanted by association fibers in the posterior piriform cortex (Haberly and Price, 1977; Neville and Haberly, 2004). Neurons within each subregion form extensive connections amongst themselves, but connections between the anterior and posterior subregions are remarkably “one-way.” Neurons from the anterior piriform cortex form extensive connections with neurons in the posterior piriform cortex, but few neurons from posterior piriform cortex project back to the anterior piriform cortex (Haberly, 2001; Neville and Haberly, 2004).

The gustatory system is responsible for detecting and identifying specific chemicals (*sugars, salts, acids, alkaloids, and amino acids*) present in foods and beverages. In rodents, information related to the chemical identity and the hedonic properties of taste stimuli is carried by cranial nerves V, VII, XI and first processed by two brainstem nuclei, the nucleus of the solitary tract (NST) and parabrachial nucleus (PBN), before ascending to the gustatory thalamus (the parvicellular portion of the ventroposteromedial nucleus of the thalamus—VPMpc) (Cechetto and Saper, 1987), and ultimately reaching the gustatory cortex (GC) (Spector and Travers, 2005; Carleton et al., 2010; Maffei et al., 2012; Ohla et al., 2019; Vincis and Fontanini, 2019; Gehrlach et al., 2020). The gustatory cortex is located within the insular cortex on the lateral surface of the brain, beginning dorsal to the rhinal vein and centered around the middle cerebral artery (Allen et al., 1991; Carleton et al., 2010; Maffei et al., 2012) (Figure 1). It is divided into three cytoarchitecturally distinct subdivisions along its dorso-ventral plane: the granular, dysgranular, and agranular gustatory cortex (Allen et al., 1991; Maffei et al., 2012; Vincis and Fontanini, 2019). These subdivisions are defined by the gradual disappearance of the granular layer (i.e., layer IV) and a reorganization of the laminar structure. Where the granular gustatory cortex is identified by its traditional 6-layered neocortical architecture, the dysgranular subdivision is characterized by a progressively fading layer IV, and the agranular subdivision, being completely void of a layer IV, is defined by its tri-laminar paleocortical architecture (Cechetto and Saper, 1987; Shi and Cassell, 1998b; Maffei et al., 2012). Although differently structured, these subdivisions are highly interconnected with anatomical tracing studies identifying feedforward and feedback interconnectivity between all of the subdivisions of the gustatory cortex (Shi and Cassell, 1998b).

**Figure 2:**
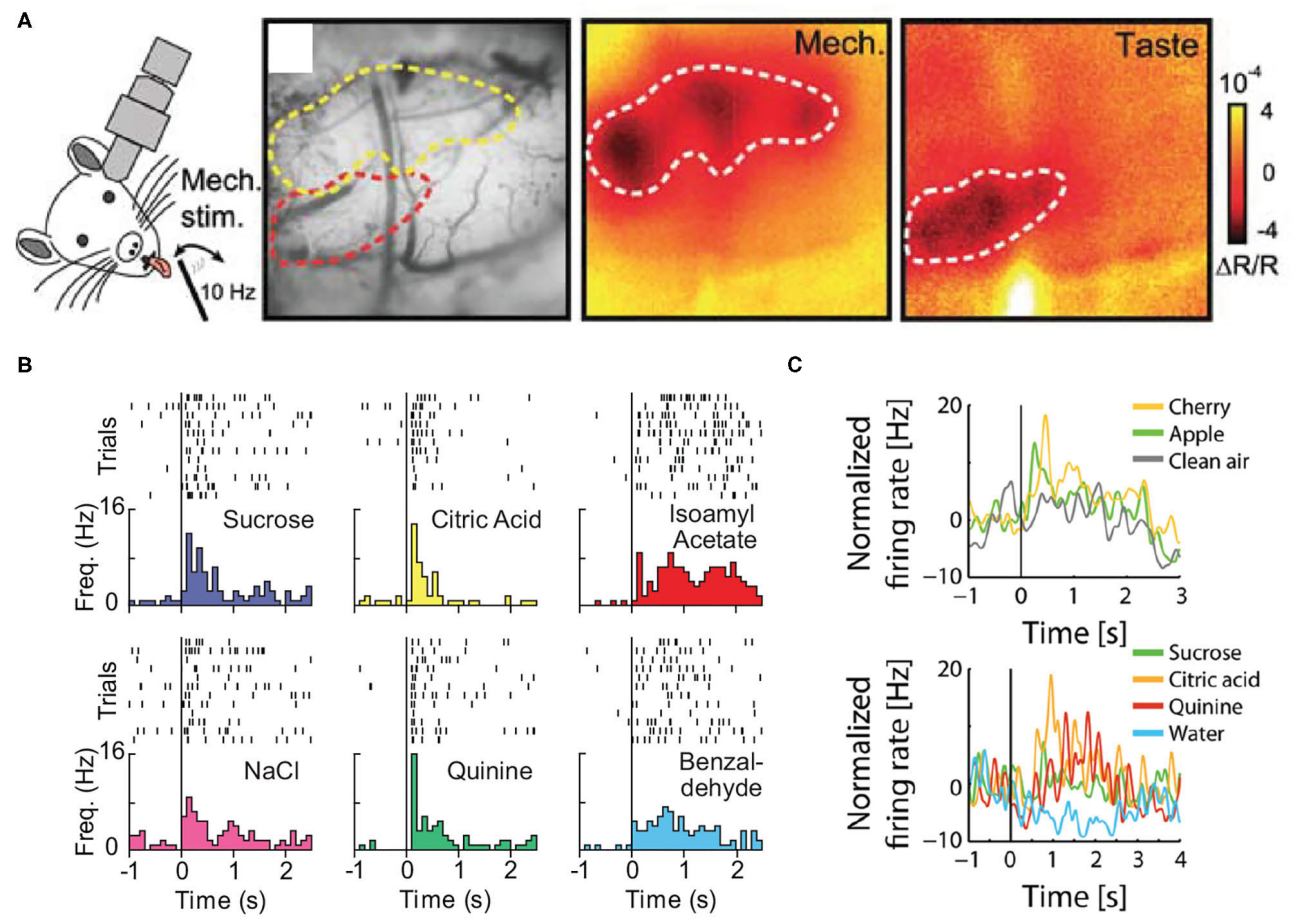
**(A)** *In vivo* intrinsic optical imaging of the oral-somatosensory cortex and gustatory cortex; note that the cortical region responding to taste stimuli is located ventral to the area activated by the tactile stimulation of the tongue. **(B)** The single-unit activity of a neuron in the gustatory cortex in response to the intraoral delivery of individual taste stimuli (sucrose, NaCl, citric acid, and quinine) and individual odors dissolved in water (isoamyl acetate and benzaldehyde). **(C)** The normalized activity of a neuron in the posterior piriform cortex in response to orthonasally presented odors (apple, cherry, and clean air) and intraorally delivered taste stimuli (sucrose, citric acid, quinine, and water). Panels adapted from **(A)** Copyright (2007) Society for Neuroscience (Accolla et al., 2007), **(B)** (Samuelsen and Fontanini, 2017), and **(C)** (Maier et al., 2012).

Somatosensation of the face and mouth relies on a number of cranial nerves to convey sensory information relative to touch, temperature, proprioception, and pain. In the brainstem, the spinal trigeminal nucleus and the principal sensory trigeminal nucleus receive somatic sensory input from cranial nerves V, VII, IX, and X, thus representing somatic sensory signals from the entire oral cavity and surface of the face (Erzurumlu and Killackey, 1979; Capra and Dessem, 1992). The oral somatic signals are then transmitted to the ventral posteromedial nucleus and the posteromedial complex of thalamus before reaching the oral-somatosensory cortex (Carvell and Simons, 1987; Spreafico et al., 1987; Liao and Yen, 2008; Ohno et al., 2012). Anatomical and functional studies confirmed that the cortical area representing somatosensory inputs from the tongue and the intraoral region are located on the most lateral portion of the somatosensory cortex, rostral to the nose and the whisker barrel fields (Remple et al., 2003; Song et al., 2018; Mayrhofer et al., 2019) and immediately dorsal to the gustatory cortex (Accolla et al., 2007; Nakamura et al., 2015) (Figure 2A). The overall topography and connectivity of the primary (SI) and secondary (SII) somatosensory cortical regions has been mapped precisely in rodents (Chapin and Lin, 1984; Liao and Yen, 2008). Historically, transmission of somatosensory signals from the periphery was thought to follow a hierarchical scheme, in which sensory information is processed sequentially from the thalamus to SI and then to the “higher-order” SII (Koralek et al., 1990; Fabri and Burton, 1991; Brett-Green et al., 2003, 2004; Jones, 2012). However, evidence from a number of rodent studies supports an equivalent hierarchy between SI and SII, where somatic inputs are processed in parallel rather than serially (Carvell and Simons, 1986; Heppelmann et al., 2001; Menzel and Barth, 2005; Liao and Yen, 2008). In this scenario, sensory information is rapidly transmitted to SI, but also to SII within a short latency (milliseconds) (Kwegyir-Afful and Keller, 2004; Benison et al., 2007; Hubatz et al., 2020). For these reasons, we define both SI and SII as *oral-somatosensory cortex*, although we will highlight important differences between the two areas when especially pertinent.

The confluence of the olfactory, gustatory, and oral-somatosensory pathways supports the hypothesis that convergence and integration of all three intraoral signals occurs at the level of the primary sensory cortical regions (Small, 2012). In rodents, these cortical areas are located on the ventro-lateral surface of the brain, with the oral-somatosensory cortex most dorsal, the piriform cortex most ventral, and the gustatory cortex sandwiched in between (Figure 1). Specifically, the dorsal component of the rodent's gustatory cortex, the granular area, lies just ventral to somatosensory areas (SI and SII) representing oral regions (Yamamoto et al., 1981; Kosar et al., 1986; Cechetto and Saper, 1987; Accolla et al., 2007; Nakamura et al., 2015), while its most ventral component, the agranular area, is located immediately dorsal to the piriform cortex (Carleton et al., 2010; Maffei et al., 2012). In addition to their physical proximity, their substantial corticocortical connectivity provides further anatomical evidence for interactions when processing intraoral signals. Both the anterior and posterior piriform cortex form dense reciprocal connections with the agranular portion of the gustatory cortex (Krushel and van Der Kooy, 1988; Datiche and Cattarelli, 1996; Shi and Cassell, 1998a,b; Johnson et al., 2000; Sewards and Sewards, 2001). Furthermore, studies by Shi and Cassel provided detailed analyses of the neural efferents from the different subdivisions of the gustatory cortex demonstrating that corticocortical projections from the granular/dysgranular gustatory cortex project to the oral-somatosensory cortex (both SI and SII) (Shi and Cassell, 1998b). Another of their studies showed that these corticocortical connections are not unidirectional, with the different divisions of the gustatory cortex receiving projections from the somatosensory cortex (Shi and Cassell, 1998a). To the best of our knowledge, we are unaware of any anatomical studies showing direct corticocortical projections from either the anterior or posterior piriform cortex to the intraoral field of the somatosensory cortex. However, in a recent anatomical tracing experiment using pseudo rabies virus, Wang et al. (2020) found that a subset of neurons in the somatosensory cortex projects to the piriform cortex, preferentially targeting the posterior region (Wang et al., 2020). Whether these monosynaptic connections originate specifically from the oral-somatosensory cortex remains unclear.

## 3. Unimodal Processing

Traditionally, most sensory neuroscience studies employ unimodal stimuli to investigate sensory processing. These findings provide the foundation for probing the circuit mechanisms underlying the multimodal processing of intraoral stimuli subtending the perception of flavor. In this section, we discuss experimental findings from rodent studies describing how the features of unimodal stimuli are represented by the piriform, gustatory, and somatosensory cortical regions, focusing on intraoral stimulation where available.

Most studies investigating the cortical processing of olfactory signals have focused on understanding how orthonasal odors are represented by the piriform cortex (Wilson and Sullivan, 2011). Furthermore, due in part to the density of input from the main olfactory bulb, these studies primarily focused on the anterior piriform cortex (Wilson, 1998, 2000, 2003; Rennaker et al., 2007; Zhan and Luo, 2010; Miura et al., 2012; Bolding and Franks, 2017; Iurilli and Datta, 2017). Multiple experimental approaches in rodents, including odor-evoked immediate early gene expression, imaging, and electrophysiological recordings, show that responses to odors are spatially distributed across ensembles of neurons in the piriform cortex without regard to chemotopy (Illig and Haberly, 2003; Rennaker et al., 2007; Stettler and Axel, 2009; Roland et al., 2017; Pashkovski et al., 2020). Extracellular recordings in anaesthetized and alert rodents revealed that neurons in the anterior piriform cortex represent the chemical identity of odors (Wilson, 1998, 2000, 2003; Rennaker et al., 2007; Zhan and Luo, 2010; Miura et al., 2012), with ensembles of activated neurons capable of accurately classifying odors within the first 100ms of inhalation (Bolding and Franks, 2017; Iurilli and Datta, 2017; Blazing and Franks, 2020). Furthermore, odor-evoked activity in the anterior piriform cortex represents a mixture of multiple odors as distinct from its individual odor components (Wilson, 2000, 2003; Kadohisa and Wilson, 2006; Wilson et al., 2020). Where neurons in the anterior piriform cortex represent odor identity (e.g., orange), neurons in the posterior piriform cortex represent the general quality/category of an odor (e.g., citrus) (Litaudon et al., 2003; Kadohisa and Wilson, 2006; Wilson et al., 2020) and may be involved in associating odors with stimulus values (Calu et al., 2007). The development of multiphoton imaging has confirmed many of the coding properties of piriform cortex described by single-unit electrophysiology studies (Stettler and Axel, 2009; Roland et al., 2017; Pashkovski et al., 2020). Recently, a study by Pashkovski et al. (2020) employed multi-photon imaging of the posterior piriform cortex in “wakeful” mice to demonstrate that the chemical representation of odors provided by the olfactory bulb is transformed to cluster together representations of related odors in layer 3 (and in layer 2 to a lesser extent) of the piriform cortex. While these studies provide elemental insight into the neural processing of the piriform cortex, the lack of studies examining retronasal olfaction overlooks a key aspect of flavor perception.

Retronasal olfaction is a fundamental component of flavor perception (Murphy et al., 1977; Rozin, 1982; Lim and Johnson, 2011) and, to our knowledge, only one study has examined how neurons in the piriform cortex represent retronasal odors in behaving rats (Maier, 2017). In this study, Maier reported that the intraoral delivery of odors dissolved in water elicited variable and extended dynamic responses over a 2 s time course in the posterior piriform cortex. In a subset of recordings, they also probed how odor-evoked responses differed when delivered either orthonasally or retronasally. While they found that some individual neurons in the posterior piriform cortex showed differences between the mode of delivery, there was no difference at the population level. Although the relatively small data set (13 neurons making 26 neuron-odor pairs) precludes definitive interpretation, these findings reveal the intricacy of olfactory processing and underscores the necessity of investigating the cortical mechanisms underlying the multimodal processing of flavor.

Over the last 40 years, multiple studies have investigated the taste response profile of cortical neurons. While it is important to highlight that taste-responsive neurons in the gustatory cortex are often multimodal (see the section 4), these studies showed that neurons in the gustatory cortex represent the identity and hedonic value of taste stimuli. Neurophysiological data obtained from extracellular recordings in anaesthetized and alert rodents highlight the presence of both narrowly-tuned neurons (those modulated by one taste quality) and broadly-tuned neurons (those modulated by multiple taste qualities) (Yamamoto et al., 1981; Kosar et al., 1986; Ogawa et al., 1992; Katz et al., 2001; Stapleton et al., 2006; Jezzini et al., 2013; Levitan et al., 2019; Bouaichi and Vincis, 2020; Dikecligil et al., 2020), with the latter being the majority in awake conditions (Katz et al., 2001; Stapleton et al., 2006; Samuelsen et al., 2012, 2013; Jezzini et al., 2013; Levitan et al., 2019; Bouaichi and Vincis, 2020). Studies in alert rodents, receiving taste stimuli either via an intraoral cannula (IOC) or by licking a spout, emphasized the importance of the temporal dynamics of taste-evoked activity. For example, the intraoral delivery of taste stimuli evokes different epochs in firing rates during the first 2.5 s. In this context, the neural activity first represents the presence (~0–250 ms), then the identity (~250–750 ms), and finally the hedonic value of taste stimuli (Katz et al., 2001; Fontanini and Katz, 2006; Jones et al., 2007; Grossman et al., 2008; Piette et al., 2012; Sadacca et al., 2012; Jezzini et al., 2013; Samuelsen et al., 2013; Levitan et al., 2019; Mukherjee et al., 2019). In addition, studies in which rodents lick a spout to receive taste stimuli revealed additional complex and rich temporal dynamics related to licking rhythmicity in the gustatory cortex (see the section 4) (Stapleton et al., 2006; Gutierrez et al., 2010; Bouaichi and Vincis, 2020).

Beyond the temporal properties of single neurons, multiple groups have investigated whether taste responses in the gustatory cortex are spatially organized in a chemotopic fashion. Optical imaging studies in anesthetized rodents have reported discrepant findings. One fluorescent-imaging study, using a calcium-sensitive dye (Oregon Green) in anaesthetized mice (Chen et al., 2011), reported that the superficial layers of the gustatory cortex are organized in a strict chemotopic map; where taste stimuli activated well-separated clusters (up to 1.5 mm apart) of narrowly tuned neurons (*hot spots*), interposed by large cortical areas void of activity. Conversely, a study in anaesthetized rats using intrinsic imaging (Accolla et al., 2007) and one in anaesthetized mice using calcium imaging (GCaMP6s) (Fletcher et al., 2017) showed an extensive degree of overlap in the response to different taste qualities in the gustatory cortex; where the spatial organization of taste responses showed a continuous distribution lacking discrete anatomical clustering and no unresponsive areas. These latter observations were confirmed by calcium imaging (GCaMP6s) studies in awake mice (Livneh et al., 2017; Chen et al., 2021), which reported that taste responses in the superficial layers of the gustatory cortex show neither a large-scale (millimeters) nor a fine-scale (tens of micrometers) topographical organization. Few experiments have investigated the intracortical circuitry of the gustatory cortex. Although these studies denote potential differences in responsiveness across subdivisions (Kosar et al., 1986; Ogawa et al., 1992) (but see Livneh et al., 2017), across layers, and between neuron types (classified based on physiological properties) (Yokota et al., 2011; Dikecligil et al., 2020), significantly more work is required to further address the circuit properties of the gustatory cortex.

Compared to the piriform and gustatory cortical regions, less information is available on the response properties of the oral-somatosensory cortex. Indeed, while the whisker barrel field (i.e., region of the somatosensory cortex that processes tactile signals from the whisker pad) has been extensively studied, few experiments have investigated the cortical organization of somatic inputs from the oral cavity. Early electrophysiological studies in anesthetized rats provided the first experimental evidence of intraoral tactile-evoked neural activity within the lateral somatosensory cortex (Welker, 1971; Yamamoto et al., 1981; Chapin and Lin, 1984; Kosar et al., 1986). Of particular interest is a study performed by Remple et al. (2003), where they used a microelectrode mapping technique to carefully investigate the topographical organization of the rat's lateral somatosensory cortex in response to somatic stimulation of multiple intraoral structures. They observed a somatotopic organization in which the cortical areas responding to the dental pulp of lower and upper incisors flanked the region receiving inputs from the tongue/inner mouth. Interestingly, these regions extend along the anterior-posterior axes and, for the most part, are located just dorsal to the gustatory cortex. These *in vivo* observations were later confirmed by multiple research groups. In 2007, Accolla et al. performed intrinsic imaging in the lateral cortical region encompassing both the rat's gustatory cortex and oral-somatosensory cortex (Accolla et al., 2007). While their main focus was on taste-evoked responses in the gustatory cortex, they also performed control experiments probing cortical activity in response to tactile stimulation of the tongue. Similar to the findings of Remple et al., Accolla and colleagues (Figure 2A) showed that the tongue cortical field is located in the oral-somatosensory cortex, just dorsal to the gustatory cortex. More recently, two studies in anesthetized rats, provided deeper insight into the representation of somatic sensory signals by the oral-somatosensory cortex. Nakamura et al. (2015) performed extracellular recordings and imaging of voltage sensitive dye to probe the neural responsiveness and topographical organization of the somatosensory cortex during the electrical stimulation of multiple extraoral and intraoral regions (Nakamura et al., 2015). Where Clemens et al. (2018) used whole-cell recordings to examine post-synaptic responses to tactile and thermal stimuli in the oral-somatosensory cortex (Clemens et al., 2018). These studies expanded upon the data obtained by Remple et al. and verified the location of the oral-somatosensory region, with the tongue field located in between the mandibular incisor and molar responsive areas.

## 4. Multimodal Processing

Traditional theories of multisensory integration propose that information from different sensory modalities is first isolated and processed by the primary sensory cortical regions before being integrated by higher-order areas (Felleman and Van Essen, 1991). This hierarchical view is being challenged by recent findings showing that corticocortical connections between sensory areas modulate responses to multimodal stimuli at the single-unit level in visual cortex (Iurilli et al., 2012; Ibrahim et al., 2016; Meijer et al., 2017; Chanauria et al., 2019), auditory cortex (Atilgan et al., 2018), and somatosensory cortex (Sieben et al., 2013; Stehberg et al., 2014; Bieler et al., 2017). In this section, we discuss experimental findings, as well as the gaps in the current knowledge, in effort to elucidate the neural mechanisms underlying the multimodal processing of intraoral stimuli.

The vast majority of knowledge pertaining to cortical multimodal processing of intraoral stimuli comes from experiments focused on the gustatory cortex. Experimental evidence from electrophysiological and optical imaging studies shows that neurons in the gustatory cortex represent non-gustatory multimodal stimuli experienced before and/or during sampling (Yamamoto et al., 1981; Kosar et al., 1986; Katz et al., 2001; Samuelsen et al., 2012, 2013; Vincis and Fontanini, 2016; Livneh et al., 2017; Maier, 2017; Samuelsen and Fontanini, 2017; Chen et al., 2021). Of particular relevance for this review are the studies investigating the representation of intraoral olfactory (retronasal) and somatosensory stimuli by neurons in the gustatory cortex. Two recent studies using multielectrode recordings in behaving rats showed that neurons in the gustatory cortex are modulated by the intraoral delivery of tasteless odors dissolved in water (Maier, 2017; Samuelsen and Fontanini, 2017) (Figure 2B). Furthermore, Samuelsen and Fontanini showed that while most neurons in the gustatory cortex responded exclusively to either odor or taste stimuli (unimodal), a significant proportion of neurons responded to both chemosensory modalities (tastes and odors; bimodal) (Samuelsen and Fontanini, 2017). One potential caveat is represented by the liquid nature of the odors delivered into the mouth. As a consequence, rather than representing olfactory signals, the responses in the gustatory cortex might also reflect somatosensory and/or taste-related activity. However, Samuelsen and Fontanini demonstrated that the odor-evoked activity in the gustatory cortex was linked with respiration and depended upon olfactory inputs (Samuelsen and Fontanini, 2017). These studies provide single-unit evidence describing the multisensory nature of the gustatory cortex, but many questions remain as to its involvement in processing multimodal chemosensory signals. For example, it is still unclear how neurons in the gustatory cortex represent an odor-taste mixtures compared to the mixture's individual components or whether specific subsets of neurons solely respond to unimodal or multimodal signals.

Many studies, in both anesthetized and alert rodents, show that neurons in the gustatory cortex respond to somatosensory tactile stimulation of the tongue and oral cavity (Yamamoto et al., 1981, 1988; Kosar et al., 1986; Katz et al., 2001; Stapleton et al., 2006; Gutierrez et al., 2010; Bouaichi and Vincis, 2020; Dikecligil et al., 2020). In awake behaving rodents, when taste stimuli are delivered directly into the mouth via IOCs, somatosensory responses emerge as fast and phasic changes in neural activity within 200ms following fluid delivery (Katz et al., 2001). Moreover, neurons in the gustatory cortex exhibit somatosensory-evoked activity when taste-delivery is contingent upon licking a spout (Stapleton et al., 2006; Gutierrez et al., 2010; Bouaichi and Vincis, 2020; Dikecligil et al., 2020). In this condition, the vast majority of neurons exhibit spiking activity entrained to licking at rates between 6 to 12 Hz. However, it is important to highlight that it is still unknown whether this rhythmic activity is merely the result of tactile stimulation of the tongue (following its contact with licking spout) or also features a motor component. Nevertheless, it is noteworthy to mention that while not all licking-coherent neurons respond to gustatory information, a significant subset of neurons with spiking activity correlated with licks accurately represents taste signals (Bouaichi and Vincis, 2020; Dikecligil et al., 2020).

Fewer studies have examined how neurons in the gustatory cortex respond to other intraoral somatosensory features, such as variations in temperature and texture. Although pioneering work in anesthetized rats indicates that thermal changes of fluid solutions seems to modulate the activity of a subset of neurons (Yamamoto et al., 1981, 1988; Kosar et al., 1986), we are unaware of any studies examining the effects of temperature or texture in behaving rodents. To the best of our knowledge, the only studies examining the neural correlates evoked by these intra-oral somatosensory features in behaving animals are obtained from the primates insular/opercular cortex (Verhagen et al., 2004; Kadohisa et al., 2005). These data implicate the gustatory cortex as a key region for the multimodal processing of taste with thermal and texture signals, but many questions remain. For instance, it is unknown if and how neurons in the gustatory cortex of alert rodents are also modulated by intraoral thermal and texture stimuli. Moreover, no evidence is available on the role that temperature and texture play in shaping the chemosensory response profile of neurons in the gustatory cortex.

Although there are only a handful of electrophysiology experiments investigating convergence of olfactory and gustatory signals in the piriform cortex, their results offer keen insights into the possible corticocortical processes underlying multisensory integration. These studies found that subsets of neurons in the posterior piriform cortex selectively represent orthonasal odor stimuli and intraoral taste stimuli (Maier et al., 2012, 2015) (Figure 2C). Furthermore, simultaneous recordings in the posterior piriform cortex and gustatory cortex revealed that taste-evoked activity is functionally correlated between the cortical regions (Maier et al., 2015). Arguably the most interesting finding from these studies is that optogenetic perturbation of the gustatory cortex significantly decreased taste-evoked activity and modulated odor-evoked responses in the posterior piriform cortex (Maier et al., 2015). These findings suggest a functional relationship between the posterior piriform cortex and gustatory cortex for processing unimodal chemosensory signals. Future studies are needed to better understand the role of these corticocortical circuits for the integration and processing multimodal chemosensory signals.

To our knowledge, only one electrophysiology study has examined whether the oral-somatosensory cortex represents gustatory signals. Clemens and colleagues performed *in vivo* whole-cell recordings in the rat's oral-somatosensory cortex (Clemens et al., 2018). In addition to probing post-synaptic responses to tactile and thermal stimuli, they investigated whether taste information was represented by neurons in the oral-somatosensory cortex. Their analysis of subthreshold membrane responses to water and two different taste stimuli (sucrose and quinine), revealed that the oral-somatosensory cortex is not robustly sensitive to sweet or bitter taste (Clemens et al., 2018). While this study suggests that gustatory signals are not represented, additional experiments probing taste-evoked activity in response to a wider variety of gustatory stimuli are needed to better elucidate taste responsiveness in the oral-somatosensory cortex.

## 5. Final Remarks

Throughout this review, we highlighted the evidence from rodent studies indicating that the primary sensory cortical regions that process information arising from within the mouth play a key role in the processing of flavor. Specifically, we reviewed neurophysiological findings of how neurons of the three cortical regions represent and process the unimodal and multimodal information related to flavor as well as their corticocortical connectivity. While it is clear more work is needed, the emerging picture is that, the piriform, gustatory, and somatosensory cortical regions do not function solely as independent areas. Rather, they act together as an intraoral cortical hub—with the gustatory cortex representing the anatomical and functional core—that simultaneously receives and processes intraoral multimodal sensory signals. Of course, these cortical areas are not the sole brain regions affecting the perception of flavor. While outside the scope of this review, many higher-order regions are known to process salient information relevant to flavor, including the orbitofrontal cortex (Rolls and Baylis, 1994; Lipton et al., 1999; Kadohisa et al., 2005; Roesch et al., 2007), the amygdala (Grossman et al., 2008; Piette et al., 2012; Sadacca et al., 2012), and the mediodorsal thalamus (Courtiol and Wilson, 2014, 2016; Pelzer et al., 2017; Fredericksen et al., 2019). Additionally, subcortical and brainstem areas integrate bottom-up as well as cortico-fugal top-down inputs salient for taste-mouthfeel and taste-smell interactions (Beidler, 1954; Nagaki et al., 1964; Sato, 1967; Van Buskirk and Erickson, 1977; Di Lorenzo and Garcia, 1985; Ogawa et al., 1988; Travers and Norgren, 1995; Verhagen et al., 2003; Breza et al., 2006; Wilson and Lemon, 2013; Escanilla et al., 2015; Li and Lemon, 2019). Regardless, the experimental evidence discussed here indicates that the cortex is likely the first site of convergence across the bottom-up pathways of the three main sensory components of flavor.

To begin to understand why we choose the foods we eat, experiments must start to elucidate the neural mechanisms underlying the integration of the intraoral senses. Thus, it is imperative to embrace the complexity of the sensory features of foods and beverages, and design experiments—in behaving animals—to probe the behavioral and neurophysiological correlates evoked by the components of intraoral stimuli as well as their associations. For example, how are the sensory signals from the three intraoral modalities represented in the piriform cortex and oral-somatosensory cortex of behaving animals? Are corticocortical connections necessary for the integration and processing of multimodal intraoral signals? How does experience with intraoral odor-taste mixtures shape cortical processing and influence consummatory behaviors and food choices? Do changes in the temperature and/or texture of a food object significantly alter the neural representation and behavioral salience of its associated odors and tastes? These are just some of the questions that can guide future experimental endeavors to progress toward a better understanding of the neural and behavioral correlates driving the perception of flavor.

## Author Contributions

RV and CS carried out study conceptualization and wrote the manuscript. All authors contributed to the article and approved the submitted version.

## Funding

This work has been supported by National Institute on Deafness and Other Communication Disorders Grant R21-DC016714 (RV) and R01-DC018273 (CS).

## Conflict of Interest

The authors declare that the research was conducted in the absence of any commercial or financial relationships that could be construed as a potential conflict of interest.

## Publisher's Note

All claims expressed in this article are solely those of the authors and do not necessarily represent those of their affiliated organizations, or those of the publisher, the editors and the reviewers. Any product that may be evaluated in this article, or claim that may be made by its manufacturer, is not guaranteed or endorsed by the publisher.
